# Gamma-secretase subunits associate in intracellular membrane compartments in *Arabidopsis thaliana*

**DOI:** 10.1093/jxb/eru147

**Published:** 2014-04-10

**Authors:** Michalina Smolarkiewicz, Tomasz Skrzypczak, Michał Michalak, Krzysztof Leśniewicz, J. Ross Walker, Gwyneth Ingram, Przemysław Wojtaszek

**Affiliations:** ^1^Department of Molecular and Cellular Biology, Adam Mickiewicz University, Umultowska 89, 61–614 Poznań, Poland; ^2^Institute of Molecular Plant Sciences, University of Edinburgh, King’s Buildings, Mayfield Rd, Edinburgh EH9 3JH, UK; ^3^UMR 5667 CNRS-INRA-ENSL-UCB Lyon I, Reproduction et Développement des Plantes, ENS Lyon, 46, Allée d’Italie, 69364 LYON Cedex 07, France

**Keywords:** Authophagy, complex assembly, endomembrane system, gamma-secretase, intramembrane protease, localization, presenilin, senescence, vesicular trafficking.

## Abstract

Gamma-secretase is a multisubunit complex with intramembrane proteolytic activity. In humans it was identified in genetic screens of patients suffering from familial forms of Alzheimer’s disease, and since then it was shown to mediate cleavage of more than 80 substrates, including amyloid precursor protein or Notch receptor. Moreover, in animals, γ-secretase was shown to be involved in regulation of a wide range of cellular events, including cell signalling, regulation of endocytosis of membrane proteins, their trafficking, and degradation. Here we show that genes coding for γ-secretase homologues are present in plant genomes. Also, amino acid motifs crucial for γ-secretase activity are conserved in plants. Moreover, all γ-secretase subunits: PS1/PS2, APH-1, PEN-2, and NCT colocalize and interact with each other in *Arabidopsis thaliana* protoplasts. The intracellular localization of γ-secretase subunits in *Arabidopsis* protoplasts revealed a distribution in endomembrane system compartments that is consistent with data from animal studies. Together, our data may be considered as a starting point for analysis of γ-secretase in plants.

## Summary

In animals, gamma-secretase is crucial for regulation of important developmental processes. Here, we show that genes encoding components of this complex are present in *Arabidopsis*, and the complex undergoes complicated assembly in the endomembrane system. This complex may have some role in autophagy.

## Introduction

Gamma-secretase is an I-CLiP (intramembrane-cleaving protease), which is involved in the process of regulated intramembrane proteolysis (RIP), in which membrane-anchored protein fragments are released by proteolytic cleavage (reviewed in Sannerud and Annaert, 2009). A catalytic core protein, presenilin (PS), and three other non-catalytic subunits are required for its activity. Nicastrin (NCT) promotes the maturation and proper trafficking of other complex components and is proposed to be involved in substrate recognition. The two remaining components, PEN-2 (presenilin enhancer 2) and APH-1 (anterior pharynx-defective 1), are most likely involved in complex assembly, stabilization, and trafficking (reviewed in Parks and Curtis, 2007). In humans, presenilin was identified in genetic screens of patients suffering from familial forms of Alzheimer’s disease (Sherrington *et al.*, 1995 ). Mutations in any of two presenilins (PS1 and PS2) lead to improper cleavage of amyloid precursor protein (APP) resulting in β-amyloid accumulation and progressive neurodegeneration. Beside APP more than 80 substrates for γ-secretase have since been identified (listed in Haapasalo and Kovacs, 2011). One of the most intensively studied is the Notch receptor, a key regulator of many signalling pathways (reviewed in Fortini, 2012). Other γ-secretase substrates can act as transcription factors or repressor proteins regulating gene expression (Lal and Caplan, 2011). Cleavage can also be used as a signal for degradation of transmembrane protein fragments and the maintenance of so-called ‘membrane proteostasis’ (Lemberg, 2011; Lichtenthaler *et al.*, 2011). Therefore γ-secretase-mediated cleavage is proposed to regulate a wide range of cellular events (Haapasalo and Kovacs, 2011).

Despite many years of intensive studies, γ-secretase complex assembly and maturation as well as the precise localization of its active form are still the subject of some controversy (St George-Hyslop and Fraser, 2012). Many lines of evidence show that γ-secretase subunits localize to almost all compartments of the cellular endomembrane system, including the endoplasmic reticulum (ER), pre-Golgi, Golgi, post-Golgi, plasma membrane, and various types of endosomes, lysosomes, and phagosomes (Annaert *et al.*, 1999; Pasternak *et al.*, 2003; Réchards *et al.*, 2003; Jutras *et al.*, 2005; Fukumori *et al.*, 2006; Dries and Yu, 2008; Fassler *et al.*, 2010; Smolarkiewicz *et al.*, 2013). Interestingly, although subunits of γ-secretase are abundant in the ER and Golgi, proteolytic activity of the complex seems to be restricted to the cell surface and endosomal compartments (Dries and Yu, 2008; Kaether *et al.*, 2006). These data are consistent with recent studies indicating that only mature and properly folded γ-secretase complex is able to exit the ER. This process is probably controlled by proteins recognizing exposed ER-retention signals in unfolded and unassembled γ-secretase subunits (Kaether *et al.*, 2007). To date, several novel ER retention signals have been identified in γ-secretase subunits including sequences localized at the C-terminus and in the fourth transmembrane domain (TMD4) of presenilin 1 (Kaether *et al.*, 2004; Fassler *et al.*, 2011), in the TMD1 of PEN-2 protein (Kaether *et al.*, 2007), and in the TMD of nicastrin (Spasic *et al.*, 2007). After complex assembly, ER retention signals are masked, and ER export is therefore possible (Kaether *et al.*, 2007). In addition, a growing body of data shows that in animals γ-secretase or presenilin itself may not only be a passive vesicular cargo, but can be actively involved in the regulation of endocytosis of membrane proteins, their trafficking, and degradation (Zhang *et al.*, 2006; Repetto *et al.*, 2007; Tamboli *et al.*, 2008; Smolarkiewicz *et al.*, 2013).

Compared with intensive long-term analyses of γ-secretase in animals, almost no data on γ-secretase in non-metazoans is available. A moss, *Physcomitrella patens*, was the first, and so far the only, plant species in which a role of presenilin has been shown. Interestingly, it was chosen as a model organism owing to the absence of any known animal substrate of γ-secretase (Khandelwal *et al.*, 2007). In *Physcomitrella* homologues of all γ-secretase subunits can be found, with presenilin (PpPS) present in one copy, which also makes functional investigations more convenient. A null mutant of presenilin (*Ppps*) displayed a phenotype with visible morphological and physiological changes including abnormal growth pattern, impaired chloroplast movement, and decreased uptake of FM4-64 dye, a common endocytosis tracer. Interestingly, PpPS was unable to cleave Notch1-based substrates in PS-deficient mouse fibroblasts, but was able to restore proper proliferation rate in PS-deficient mouse embryonic fibroblasts, which is thought to be independent of γ-secretase activity.

There is also evidence that γ-secretase is present and active in the slime mould, *Dictyostelium discoideum*. Presenilins have been shown to be crucial for *Dictyostelium* cell fate determination and the regulation of phagocytosis. This suggests that γ-secretase might be far more ancient and its role extends well beyond what is known from animal studies (McMains *et al.*, 2010).

Here we present experimental evidence that all γ-secretase subunits are present in higher plants and interact with each other. Moreover, we show that in plant protoplasts homologues localize to the endomembrane system, which may be an interesting clue for further functional studies.

## Material and methods

### Sequence analysis

Identification of γ-secretase homologues was conducted by PSI-BLAST (NCBI) using the “Non-redundant protein sequences” database. Sequences of human (*Homo sapiens*; Hs) γ-secretase subunits were used as queries. Searches were restricted to *Arabidopsis thaliana* (At), *Physcomitrella patens* (Pp), *Chlamydomonas reinhardtii* (Chr), and *Dictyostelium discoideum* AX4 (Dd).

Multiple sequence alignments and the cladogram were generated using Clustal Omega (Sievers *et al.*, 2011) on sequences with following accession numbers: HsPS1: P49768.1; AtPS1: O64668; PpPS: XP_001762484.1; ChrPS: XP_001701664.1; DdPS1: XP_629693.1; HsPS2: P49810.1; AtPS2: Q9SIK7; DdPS2: XP_635158.1; HsPEN-2: Q9NZ42.1; AtPEN-2: Q9FY84; PpPEN-2: XP_001770335.1; ChrPEN-2: XP_001700868.1; DdPEN-2: Q54BR1.2; HsAPH-1: Q96BI3.1; AtAPH-1: Q8L9G7; PpAPH-1: XP_001770545.1; ChrAPH-1: XP_001693775.1; DdAPH-1: XP_647460.1, HsNCT: Q92542.2; AtNCT: Q8GUM5; PpNCT: XP_001762853.1; ChrNCT: XP_001701591.1; DdNCT: XP_637065.2

### Plant material and growth conditions

Arabidopsis thaliana ecotype Columbia (Col-0) was used as the wild type. Insertion mutant lines were obtained from the Nottingham *Arabidopsis* Stock Centre (NASC): SALK_145544 (*ps1*) and SALK_013158c (*ps2*). The presence of the T-DNA in *ps1* was confirmed using the primer P1R (gaacatcatcaagtttgttgtcacccc) in combination with a T-DNA left border primer (atttgccgatttcggaac). The presence of the wild-type allele was confirmed using the P1R primer in combination with the P1F primer (acatggagtctagtatcctcgattccc). The presence of the T-DNA in *ps2* was confirmed using the P2R primer (cgaacatcacaaggttagaagaacattgc) in combination with a T-DNA primer (above). The presence of the wild-type allele was confirmed using the P2R primer in combination with the PS2F primer (acatggatcgaaatcaaagacccag). The double mutant *ps1*/*ps2* was identified in the F2 of a cross between *ps1* and *ps2* homozygous plants.

Seeds were germinated either on half-strength MS medium (Duchefa) containing 1.5% sucrose or directly in the soil. For the starvation experiments, seedlings were germinated on half-strength MS medium with 1.5% sucrose, and transferred at day 14 to a medium containing neither nitrogen nor carbon (no N/C). Plants were grown in controlled conditions: 22 °C and 16-h-light/8-h-dark cycles, or in complete darkness. For the detached-leaf assay, second true leaves were excised from 4-week-old plants and floated on water in the wells of 12-well tissue culture dishes. Detached leaves then were placed in the dark at room temperature.

### RT-PCR

Total RNA from either 2-week-old seedlings, or 6-week-old rosette leaves or stems was isolated manually with TRI Reagent (Sigma-Aldrich) according to manufacturer’s procedure. RT-PCR was conducted using M-MLV Reverse Transcriptase (Promega). Specific primer pairs were used to amplify coding sequences of γ-secretase subunits and actin 2 (At3g18780), as an internal standard: PS1 F: atggagtctagtatcctcg, R: gaacatcatcaagtttgt; PS2 F: atggatcgaaatcaaagaccc, R: aacatcacaaggttaga; NCT F: atgggacttattcgtctt, R: gtcctgcttcccggcttttgtgatg; APH F: atgacggtcgcggcgggtat, R: ccgtgaggcgctttggttccg; PEN F: atggaggctacacggagcgacgac, R: agccaaaccagacaagccgagtgc; ACT F: atggctgaggctgatgatat, R: tcatagaaacgaaaacaaaaaggg.

### cDNA cloning

cDNAs coding for most γ-secretase subunits was obtained from RIKEN BioResource Center. The following clones were used: RAFL09-09-N23 (AtPS1); RAFL08-17-N09 (AtNCT); RAFL09-21-F15 (AtAPH-1); and RAFL19-93-N04 (AtPEN-2). The cDNA coding for AtPS2 was synthesized with M-MLV Reverse Transcriptase (Promega) from total RNA extracted manually from 6-week-old *Arabidopsis* leaves and amplified using primer pair cPS2 (F: atggatcgaaatcaaagaccc, R: gaacatcacaaggttaga).

### Vector construction

For localization and interaction studies, sequences coding for γ-secretase subunits were introduced into the pSITE expression vectors (Chakrabarty *et al.*, 2007), which utilize the Gateway® Cloning System (Invitrogen). To generate Entry Clones, sequences coding for γ-secretase subunits were amplified in two subsequent PCR reactions using Phusion® High-Fidelity DNA Polymerase (Finnzymes) with primers adding Gateway adaptor sequences. The following primers were used: gPS1 F: aaaaagcaggcttcatggagtctagtatcctcg, R: agaaagctgggtggaacatcatcaagtttgt; gPS2 F: aaaaagcaggcttcatggatcgaaatcaaagac, R: agaaagctgggtggaacatcacaaggttaga; gNCTF: aaaaagcaggcttcatgggacttattcgtctt, R:agaaagctgggtggtcctgcttcccggcttttgtgatg; gAPH-1 F:aaaaagcaggcttcatgacggtcgcggcgggtat, R:agaaagctgggtgccgtgaggcgctttggttccg; gPEN-2 F:aaaagcaggcttcatggaggctacacggagcgacg, R: agaaagctgggtgagccaaaccagacaagcc gagtgc. Product of desired length from first reaction was eluted using Gene Elute Kit (A&A) and subjected to second amplification with UNI primer pair: F: ggggacaagtttgtacaaaaaagcaggct, R: ggggaccactttgtacaagaaagctgggt. Amplified sequences were then introduced into the pDONR207 vector by BP Reaction (Gateway, Invitrogen) according to manufacturer’s instructions. Next, coding sequences for γ-secretase subunits were cloned by LR Reaction (Gateway, Invitrogen) into pSITE_NB vectors to generate C-terminal fusions with distinct fluorescent proteins.

### Site-directed mutagenesis

To introduce the point mutation into the AtPEN-2 sequence, a PCR reaction was conducted on the appropriate Entry Clone with the SDM primers: F: aagttcggtttcgctctcgagttgccttggctttggttt, R: aaaccaaagccaaggcaactcgagagcgaaaccgaactt. The PCR reaction was followed by restrictive digestion with DpnI (Fermentas). The presence of the mutation was confirmed by sequencing. The AtPEN-2^N74L^ coding sequence was then sub-cloned by LR reaction (Gateway®, Invitrogen) into pSITE_2NB.

### Other constructs

For colocalization studies the ‘Wave_R’ set of the following marker proteins fused with mCherry were used: Wave2 (Rab F2b/ARA7); Wave6 (NIP1;1); Wave13 (VTI12) (Geldner *et al.*, 2009).

### Protoplast isolation and transfection

Protoplasts were isolated from 6-week-old leaves of *Arabidopsis thaliana*. The procedure was based on that described by Wu *et al.* (2009). Epidermis from the bottom side of leaves was peeled away using Scotch Magic Tape 3M stuck to both sides of the leaf. Approximately, one large leaf per transfection was used. Leaves were placed in Petri dishes with 20ml of the cell wall digestion enzyme solution (1.2% Cellulase R10, Serva; 0.4% Macerozyme R10, Serva; 0.4M mannitol; 20mM KCl; 20mM MES, pH 5.7) for 45min at 28 °C with gentle rotation (60rpm on a platform shaker). Protoplasts were then transferred to Falcon tubes and centrifuged (3min, 150*g* at 4 °C; Eppendorf Centrifuge 5804 R), washed twice with 15ml W5 Solution (154mM NaCl; 125mM CaCl_2_; 5mM KCl; 2mM MES pH 5.7) and immediately resuspended in 1–5ml of MMg solution (0,4M mannitol; 15mM MgCl_2_; 4mM MES pH 5.7). The final volume was adjusted to obtain the optimal concentration of 2×10^4^ cells ml^–1^. Protoplasts were incubated on ice for 15min. For transfection 100 µl of protoplasts were mixed with a maximum of 2.5–5 µg of plasmid and 110 µl of PEG solution (40% PEG 4000; 200mM mannitol; 100mM CaCl_2_) and incubated for 20min at room temperature. For colocalization studies, a total amount of 10 µg of DNA was not exceeded. 400 µl of W5 solution was added. Protoplasts were pelleted by gentle centrifugation (3min, 300*g*) and re-suspended immediately in 500 µl of W1 solution (0.5M mannitol; 20mM KCl; 4mM MES pH 5.7). Protoplasts were analysed after 8h of incubation at RT.

### Subcellular localization studies

All imaging was performed on a NIKON A1R confocal microscope. Protoplasts were observed with Plan Apo ×100 oil immersion lens. eGFP fluorescence was excited with 488nm laser line and captured using 500–550nm emission filter. mRFP and mCherry were excited with a 561nm laser line and captured using a 570–620nm emission filter. Protoplasts showing extremely high level of protein overexpression resulting in formation of fluorescent aggregates were excluded from analysis. For quantitative colocalization, sections were taken with detector settings optimized for very low background and a complete absence of channel crosstalk. Dual-colour images were acquired by sequential line switching, allowing the separation of channels by both excitation and emission. Images were captured as single optical sections. Colocalization was quantified as Pearson’s correlation coefficient (PCC) for ten independent protoplasts or for magnification of selected image with Nikon NIS-Elements. Data were processed in Nikon NIS-Elements and ImageJ MBF software. Figures were prepared in Corel PHOTO-PAINT 12.

### Protein–protein interaction studies

FLIM (Fluorescence Lifetime Imaging Microscopy) was performed using the Picoquant PicoHarp TCSPC Module in combination with a Nikon A1R confocal microscope. eGFP and mRFP were chosen as the donor::acceptor pair. Donor fluorescence was excited with a 485nm pulsed diode laser; PDL 800-D; 40mHz. The excitation light was directly coupled into the microscope and focused onto the sample using a Plan Apo ×100 oil immersion objective lens. Photons were detected using a SPAD detector module. eGFP emission was selected using 520/35nm filter. Images with a frame size of 256×256 pixels were acquired. Data analysis was performed with Picoquant’s Symphotime software. From the intensity images, complete fluorescence lifetime decays were calculated per pixel for whole images as well as for selected fragments, and fitted using a double (for donor-only samples) or triple exponential decay model (donor::acceptor samples). For FRET analysis the fluorescence lifetime of one component was fixed to the value found for native donor (T_D_). χ^2^ of 1 was considered as a perfect fit. The FRET efficiency (E) was calculated as E=1−τ_DA_/τ_D_×100%, where τ_D_ is the fluorescence lifetime of the donor in the absence of acceptor and τ_DA_ that of the donor in the presence of acceptor (Llères *et al.*, 2007; Russinova *et al.*, 2004).

## Results

### Genes coding for γ-secretase subunits are present in the *Arabidopsis thaliana* genome

**Fig. 1.**
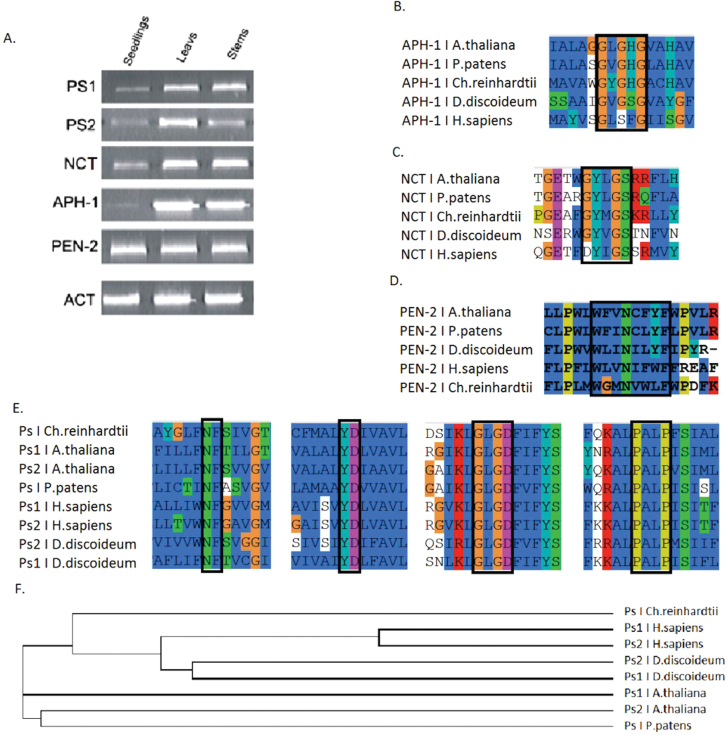
Expression and sequence analysis of γ-secretase subunits. (A) Transcripts for γ-secretase subunits could be detected in different organs of *Arabidopsis thaliana*. Reverse transcription was conducted with primers specific to each subunit. mRNA was isolated from diverse organs: seedlings, leaves, and stems. Actin 2 was used as internal standard. (B–E) Sequence alignments of amino acid motifs known from animal studies as crucial for γ-secretase activity and complex assembly. (B) APH-1: GXXXG; (C) Nicastrin: XYXGS; (D) PEN-2: WXXNXXF; (E) Presenilins: NF, YD, GXGD, and PALP motifs; (F) Cladogram created on the basis of amino acid sequence alignment for presenilins.

To check whether genes coding for γ-secretase subunits are present in plant genomes, PSI-BLAST analysis was conducted. The sequences of human γ-secretase subunits were used as queries to search for homologues in the *Arabidopsis thaliana* genome. Sequences were selected based on highest score, identity, and similarity, and are hereafter referred to as: AtPS1 (At1g08700), AtPS2 (At2g29900), AtNCT (At3g52640), AtAPH-1 (At2g31440), and AtPEN-2 (At5g09310). To test whether these sequences are expressed at the mRNA level, a reverse transcription PCR approach was used. Transcripts of γ-secretase subunits were detected in *Arabidopsis* seedlings, leaves, and stems (Fig. 1A) indicating that identified sequences are active at transcription level.

### Motifs crucial for γ-secretase activity are conserved in plant homologues

Data available from studies of γ-secretase complexes in animals indicate that certain amino acid motifs are crucial for proteolytic activity, substrate recognition, and complex assembly (reviewed in Dries and Yu, 2008). To verify if these patterns are also conserved in other organisms, multiple sequence alignments were conducted using amino acid sequences of γ-secretase component homologues from *A. thaliana, Physcomitrella patens, Chlamydomonas reinhardtii, Dictyostelium discoideum*, and *Homo sapiens* (Fig. 1B–F). Potential homologues were identified by PSI-BLAST and similarity among investigated species in reference to *Homo sapiens* sequences is presented in Table 1.

**Table 1.** Amino acid sequence similarity between particular γ-secretase subunits in various species based on PSI-BLAST analyses. Sequences from *Homo sapiens* (Hs) were used as reference sequence (query)

	HsPS1	HsPS2	HsPEN-2	HsAPH-1	HsNCT
***A. thaliana***	42.6%	44.4%	35.3%	42.0%	38.1%
***P. patens***	42.5%	39.3%	39.3%	39.0%	39.9%
***C. reinhardtii***	38.5%	37.0%	38.0%	40.4%	31.1%
***D. discoideum***	42.2%	42.5%	55.7%	37.2%	37.6%

A GXXXG motif within TMD4 of APH-1 (Fig. 1B), which in animals was shown to be involved in complex assembly through binding to presenilin (Niimura *et al.*, 2005), is well conserved. In the complex, nicastrin is thought to participate in substrate recognition. In animals, the motif DYIGS was shown to be critical for nicastrin function (Yu *et al.*, 2000; Tomita *et al.*, 2002). In *Arabidopsis*, the motif is substituted by GYLGS, in *Chlamydomonas* by GYMGS, and in *Dictyostelium* by GYVGS (Fig. 1C). Whereas amino acid changes between I, L, M, and V should not affect the hydrophobic character of the domain, the D to G substitution could have a profound influence on its polar character. Experiments done with *Dictyostelium* (McMains *et al.*, 2010) indicated, however, that γ-secretase was still proteolytically active against animal substrates, and therefore one can conclude that this substitution did not abolish complex activity. Thirdly, a well-investigated ER retention signal in the PEN-2 sequence, WLVNIFWF, in which the N residue is particularly well conserved, (Kaether *et al.*, 2007), could also be found in *Arabidopsis* PEN-2 in the form of a WFVNCFYF sequence (Fig. 1D). Finally, the catalytic motifs of presenilins, GXGD and YD, as well as the PALP motif determining the conformation of the active site (Wolfe *et al.*, 1999; Kimberly *et al.*, 2000; Yamasaki *et al.*, 2006; Sato *et al.*, 2008; Wang *et al.*, 2006) were identified in all analysed sequences. Moreover, a short NF motif in TMD4 that was characterized as a PEN-2 binding site and ER retention signal (Fassler *et al.*, 2010) was also fully conserved (Fig. 1E). All these data clearly indicate that motifs crucial for γ-secretase assembly and activity are well conserved among evolutionary distant species; however, phylogenetic analysis of presenilins indicate that plant and animal homologues fall into two divergent clades (Fig.1F).

### Gamma-secretase subunits are localized in intracellular membranous compartments in *Arabidopsis* protoplasts

**Fig. 2.**
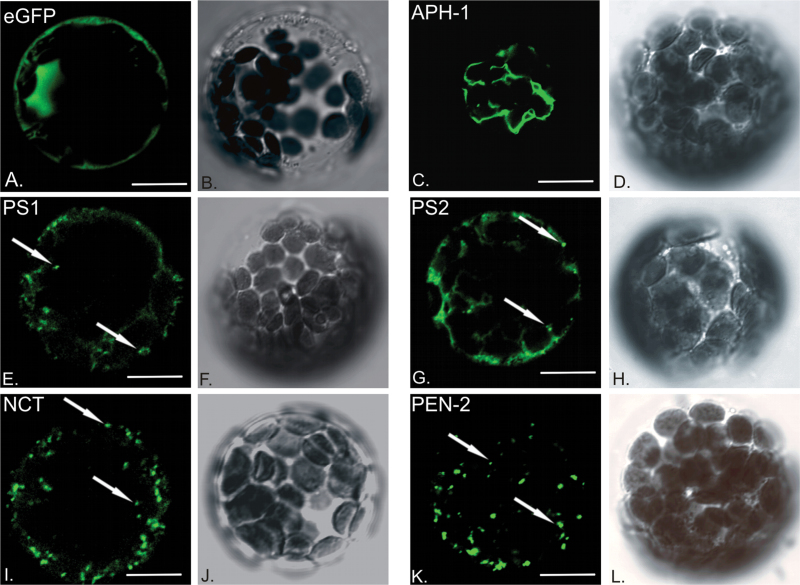
Cellular localization of γ-secretase subunits. Genetic constructs coding for γ-secretase subunits fused with eGFP were introduced to *Arabidopsis thaliana* leaf mesophyll protoplasts for transient expression and analysed with confocal microscope. (A) eGFP fluorescence alone was used as control; (C) AtAPH-1–GFP signal is visible in reticular structures; (E) AtPS1–GFP and (G) AtPS2–GFP signal marks vesicular compartments and reticular structures; (I) AtNCT–GFP and (K) AtPEN-2–GFP fluorescence visible mostly in vesicles. Transmitted light images of respective cells are presented next to fluorescent sections (B, D, F, H, J, L). White arrows point to exemplary vesicles. Bar, 10 µm.

Gamma-secretase in animals has been shown to be largely localized in the ER and Golgi. However, the pool of the proteolytically active complex is localized in vesicles and at the plasma membrane (Annaert *et al.*, 1999; Pasternak *et al.*, 2003; Réchards *et al.*, 2003; Jutras *et al.*, 2005; Fukumori *et al.*, 2006; Dries and Yu, 2008; Fassler *et al.*, 2010). Cellular localization of plant γ-secretase subunits was analysed in *A. thaliana* leaf mesophyll protoplasts. Genetic constructs coding for particular proteins fused with green fluorescent protein (GFP) under the control of the constitutive 35S CaMV promoter were introduced into protoplasts. Fusion proteins were observed at different time points by laser scanning confocal microscopy. Fluorescent images from single optical sections of cells expressing particular fusion proteins, as well as images captured using transmitted light are presented in Fig. 2.

Clear nuclear and cytoplasmic signal could be observed when native eGFP was expressed as a control (Fig. 2A). When overexpressed in protoplasts AtAPH-1–GFP signal could be found in reticular structures (Fig. 2C). Signal for AtPS1–GFP and AtPS2–GFP was restricted to the reticular compartment (20%), vesicular structures (10%), or present in both vesicular compartment and reticular structures (70%) (Fig. 2E, G). AtNCT–GFP and AtPEN-2–GFP fluorescence appeared mostly in vesicular compartments (Fig. 2I, K). Together, these data suggest localization of plant γ-secretase subunits to intracellular membranous compartments.

**Fig. 3.**
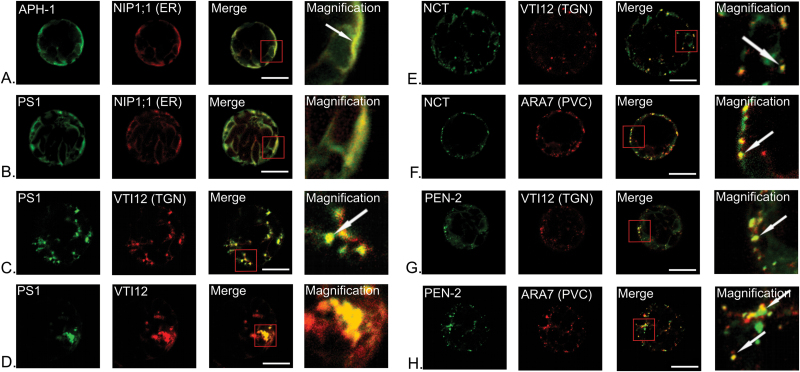
Localization of γ-secretase subunits with markers of intracellular membranous system. AtAPH-1 (A), presenilin 1 (B, C, D), nicastrin (E, F) and AtPEN-2 (G, H) were tagged with eGFP and introduced into *Arabidopsis* protoplasts simultaneously with marker proteins for different compartments: ER, NIP1;1; *trans* Golgi network (TGN), VTI12; and prevacuolar compartment (PVC), ARA7 fused with mCherry. Images for fusion with each fluorophore were captured separately and presented in different colours: eGFP in green, mCherry in red. Combined images showing colocalization of two proteins are in yellow. Magnification of areas showing pronounced colocalization is included. AtAPH-1–GFP is localized to the endoplasmic reticulum (A). AtPS1–GFP signal could be detected in ER (B) and TGN (C). However in some cells visibly enlarged compartments showing colocalization of AtPS1–GFP and VTI12–mCherry could be seen (D). AtNCT–GFP is localized to *trans* Golgi network (E) and prevacuolar compartment (F). Also PEN-2–GFP fluorescence could be detected in both: TGN (G) and PVC (H). White arrows point to exemplary colocalization areas. Bar, 10 µm.

To differentiate particular subcellular compartments, the ‘Wave’ set of genetic constructs coding for marker proteins fused with mCherry was applied (Geldner *et al.*, 2009). NIP1;1 protein (Wave6R) was used as a marker for the endoplasmic reticulum, RabF2b/ARA7 (Wave2R) to identify the prevacuolar compartment (PVC), and VTI12 (Wave13R) as a marker for the *trans* Golgi network (TGN). It should be mentioned that VTI12 protein, which is an accepted TGN marker, is also present in a subpopulation of PVC vesicles, different, however, from those ARA7-labelled. Constructs coding for γ-secretase subunits and marker proteins were introduced simultaneously into *A. thaliana* protoplasts and analysed by laser scanning confocal microscopy (Fig. 3).

Detailed analysis showed that γ-secretase subunits in plants colocalized with markers of various intracellular membranous compartments. Colocalization was quantified by Pearson’s correlation coefficient and is presented in Table 2. AtAPH-1–GFP localized to the endoplasmic reticulum (Fig. 3A). Presenilin 1 colocalized with ER marker protein (Fig. 3B) and with the TGN marker (Fig. 3C). However, it was noticed that in approximately 30% of analysed cells fluorescent aggregates could be seen (Fig. 3D). Presenilin 2 showed a very similar localization pattern to presenilin 1 (not shown). When expressed alone both AtNCT–GFP and AtPEN-2–GFP localized to vesicular compartments (see Fig. 2I, K). In our studies, AtNCT and AtPEN-2 colocalized with the VTI12–mCherry and ARA7–mCherry marker proteins (Fig. 3E–H). However, in each case, colocalization was only observed in a subset of vesicular compartments suggesting that AtNCT and AtPEN-2 are present in only a proportion of TGN and PVC, or possibly in intermediate compartment shuttling between the two.

**Table 2.** Colocalization of γ-secretase subunits with marker proteins of particular cellular compartments. Image Pearson’s correlation coefficient was calculated as an average for ten independent images. Magnification Pearson’s correlation coefficient was calculated for magnification presented on Fig. 3. SD, standard deviation

γ-secretase subunit	Marker protein	Image Pearson’s correlation coefficient±SD	Magnification Pearson’s correlation coefficient
AtAPH-1	NIP1;1 (ER)	0.93±0.04	0.97
AtPS1	NIP1;1 (ER)	0.89±0.03	0.92
AtPS1	VTI12 (TGN)	0.87±0.07	0.84
AtNCT	VTI12 (TGN)	0.80±0.03	0.70
AtNCT	ARA7 (PVC)	0.73±0.08	0.76
AtPEN-2	VTI12 (TGN)	0.85±0.03	0.87
AtPEN-2	ARA7 (PVC)	0.82±0.04	0.74

### Gamma-secretase subunits colocalize and interact with each other in *A. thaliana* protoplasts

To test if γ-secretase complexes are formed by plant homologous components it was investigated whether particular subunits could interact with each other using a FLIM-FRET approach based on the measurement of donor fluorescence lifetime. Fluorescence lifetime is the time needed for a fluorophore to return from an excited state to the ground state. Quenching of donor lifetime in the presence of a potential acceptor, compared with native donor lifetime, indicates that proteins are closely juxtaposed and may interact (Osterrieder *et al.*, 2009; Llères *et al.*, 2007; Russinova *et al.*, 2004).

**Fig. 4.**
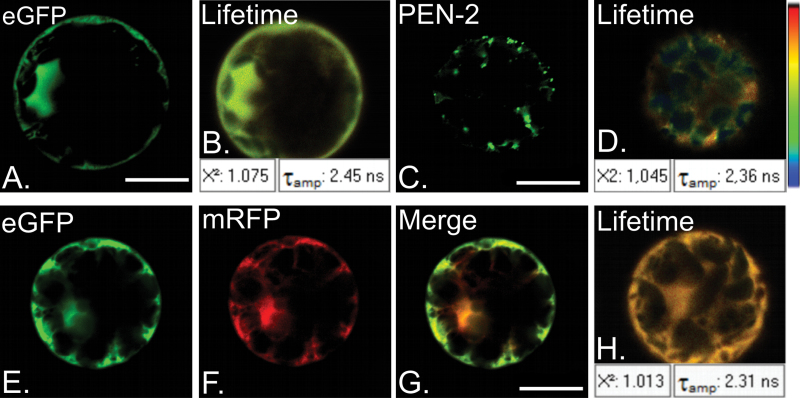
Fluorescence lifetime measurements of native donor as a control for FLIM-FRET. (A) Fluorescent signal of donor eGFP overexpressed in *Arabidopsis thaliana* protoplasts, and (B) fluorescence lifetime measurement of eGFP. Mean value of eGFP lifetime is T_amp_: 2.45 ns. (C) Fluorescent signal of eGFP fused with AtPEN-2 overexpressed in *Arabidopsis* protoplasts and (D) fluorescence lifetime measurement of AtPEN-2–GFP. Mean value of AtPEN-2–GFP lifetime is T_amp_: 2.36 ns. Fluorescent signal of donor eGFP (E), in the presence of potential acceptor mRFP (F). Merged E and F presented on (G). (H) Donor fluorescence lifetime measurement. Mean value of eGFP lifetime is T_amp_: 2.31 ns. (B, D, H) χ^2^ near 1 indicates good quality of fitting. Bar, 10 µm.

For FLIM-FRET measurements it is crucial to determine the native lifetime of the donor protein (T_D_). Constructs coding for eGFP alone, as well as fused with particular subunits of γ-secretase complex were introduced into *A. thaliana* protoplasts derived from leaves. Each lifetime measurement was repeated in at least 30 protoplasts. Fig. 4 shows representative results for free eGFP (Fig. 4A, B) and AtPEN-2–GFP (Fig. 4C, D). The average lifetime of free eGFP was estimated as T_D_GFP=2.43 ns, whereas the lifetime of eGFP fused with γ-secretase subunits was shorter and ranged from 2.34 ns for AtPEN-2–GFP to 2.39 ns for AtPS1–GFP (Table 3). As a negative control, the lifetime of eGFP as donor in the presence of mRFP as a potential acceptor (T_DA_), but in the absence of an interaction, was determined. Native eGFP and mRFP were overexpressed in *A. thaliana* protoplasts (Fig. 4E–G). The average lifetime of donor in the presence of possible acceptor (T_DA_GFP=2.31 ns, Fig. 4H) was compared with average lifetime of donor itself (T_D_GFP = 2.43 ns; see above). FRET efficiency was calculated as E=1−τ_DA_/τ_D_ = 5%. This efficiency was used to determine the threshold of FRET efficiency when an interaction is absent. Values of FRET efficiency above the threshold (5%) were considered as putative positive interactions.

**Table 3.** Colocalization of particular γ-secretase subunits, average fluorescence lifetimes of donor proteins, donor proteins in the presence of potential acceptor and FRET efficiency of distinct FRET pairs. SD, standard deviation

Donor protein	Acceptor protein	Pearson’s correlation coefficient ±SD	Donor lifetime T_D_ (ns)±SD	Donor lifetime in the presence of potential acceptor T_DA_ (ns)±SD	FRET efficiency, E_FRET_ (%)
eGFP	mRFP	0.95±0.02	2.43 ns±0.03	2.31 ns±0.04	5%
AtPS1–GFP	AtAPH-1–RFP	0.83±0.04	2.39 ns±0.02	1.94 ns±0.03	19%
AtAtPS2–GFP	AtAPH-1–RFP	0.91±0.03	2.39 ns±0.05	1.91 ns±0.09	20%
AtPEN-2–GFP	AtAPH-1–RFP	0.72±0.03	2.34 ns±0.02	2.33 ns±0.11	0.5%
AtNCT–GFP	AtAPH-1–RFP	0.84±0.03	2.36 ns±0.12	2.04 ns±0.04	14%
AtPS1–GFP	AtNCT–RFP	0.85±0.02	2.39 ns±0.02	1.93 ns±0.02	19%
AtPEN-2–GFP	AtNCT–RFP	0.81±0.05	2.34 ns±0.02	2.00 ns±0.07	11%
AtPS1–GFP	AtPEN-2–RFP	0.91±0.05	2.39 ns±0.02	1.81 ns±0.12	24%
AtPS2–GFP	AtPEN-2–RFP	0.78±0.05	2.39 ns±0.05	1.63 ns±0.22	32%
AtPEN-2^N74L^–GFP	AtPS2–RFP	0.89±0.04	2.36 ns±0.03	2.34 ns±0.03	1%

**Fig. 5.**
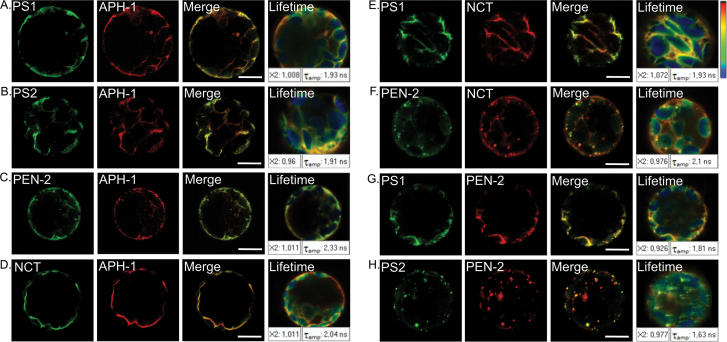
Interaction studies of γ-secretase subunits in *Arabidopsis thaliana* protoplasts. Simultaneous expression of γ-secretase subunits showed their colocalization. Fluorescent signal of particular subunit fused with GFP is presented in green and fused with RFP in red. Colocalization of γ-secretase subunits is shown in merged channel (yellow). Fluorescence lifetime measurements are presented on most-right panel and certain lifetime values are presented in white boxes (T_amp_). χ^2^ near 1 indicates good quality of fitting. Bar, 10 µm.

To verify if γ-secretase subunits interact, colocalization studies were conducted combined with FLIM measurement. Individual pairs of complex subunits fused with eGFP and mRFP were introduced simultaneously into *A. thaliana* protoplasts. First, colocalization of subunits was verified (Fig. 5 and Table 3). FLIM measurements were then performed (Fig. 5). Estimated FRET efficiencies for γ-secretase pairs are presented in Table 3.

We showed that, when co-expressed in *A. thaliana* protoplasts, γ-secretase subunits colocalize in different compartments of the intracellular membranous system. AtAPH-1 expressed with nicastrin, presenilins 1 and 2 colocalize to the reticular compartment (Fig.5A, B, D), whereas with AtPEN-2 additional minor vesicular signal could be seen (Fig. 5C). FLIM analysis showed a decrease in donor fluorescence lifetime suggesting a possible interaction between AtAPH-1 and presenilin 1 (E=19%), presenilin 2 (E=20%), and nicastrin (E=14%). Interestingly, despite strong colocalization of AtAPH-1 and AtPEN-2 no decrease in fluorescence lifetime of donor protein was observed for this pair (E=0.5%). When co-expressed, presenilin 1 and nicastrin localized mainly to reticular compartments (Fig. 5E) and showed FRET_E=19%. Interestingly, when co-expressed with AtPEN-2 nicastrin signal is detected in reticular and vesicular structures (Fig.5H). Presenilin 2 with AtPEN-2 showed localization to vesicular compartments (Fig. 5H), whereas for presenilin 1 reticular and minor vesicular fluorescence could be seen (Fig. 5G). FLIM measurements showed significant decreases in donor fluorescence lifetime indicating interactions between AtPEN-2 and AtNCT (E=11%), AtPS1 (E=24%), and AtPS2 (E=32%). In conclusion, our results show that all γ-secretase subunits, when co-expressed in *Arabidopsis* protoplasts, colocalize to a reticular or vesicular compartment, or both. Moreover, FLIM data indicate that most of the investigated proteins may interact with each other.

### An asparagine residue in TMD1 of AtPEN-2 is crucial for its vesicular localization and may be involved in interaction with presenilin 2

Our data suggest that the subcellular localization of γ-secretase subunits is highly dependent on the particular protein pair studied. Localization of AtPS2 or AtNCT to vesicular compartments could be seen only when the co-expressed proteins was AtPEN-2. This relationship was less obvious for AtPS1, as when co-expressed with AtPEN-2, it colocalized in both reticular and vesicular compartments. When expressed with AtAPH-1, reticular localization of these proteins could be observed. Interestingly, data from animal studies show that only the fully matured and properly folded complex can exit the early secretory pathway (reviewed in Dries and Yu, 2008). In animals, ER retention signals have been shown to play a pivotal role in targeting of at least presenilin, PEN-2, and NCT to destination compartments and in preventing unassembled subunits from exiting the ER/Golgi (Keather *et al.*, 2007). Keather *et al.* (2007) showed that an asparagine residue in TMD1 of the PEN-2 protein is a crucial residue for ER retention, preventing unassembled subunits from entering the secretory pathway, and is thus indispensable for PEN-2 localization.

**Fig. 6.**
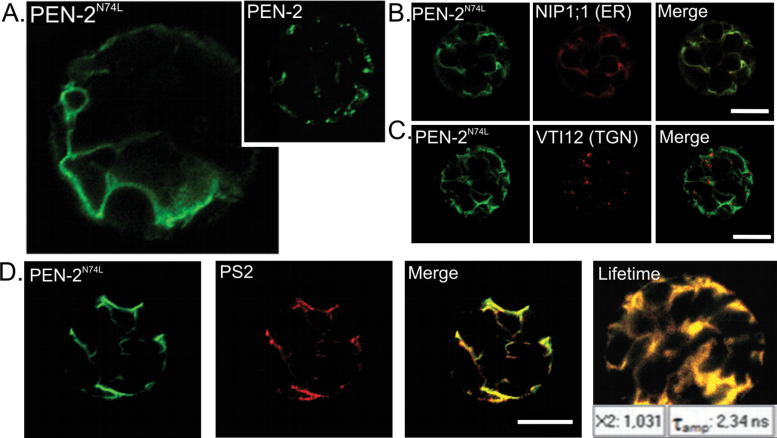
AtPEN-2^N74L^ shows reticular localization and does not interact with presenilin 1 in *Arabidopsis thaliana* protoplasts. (A) AtPEN-2^N74L^–GFP localized to reticular compartment (left) and native form of AtPEN-2–GFP localized to vesicular compartment (miniature, right). Colocalization studies with ER marker protein showed that indeed AtPEN-2^N74L^–GFP is present in endoplasmic reticulum (B). Moreover, AtPEN-2^N74L^–GFP fluorescence could no longer be detected in TGN (C) where native form of AtPEN-2 is localized (see Fig. 4G). When co-expressed with mutated AtPEN-2^N74L^, AtPS2–RFP signal was restricted to reticular compartment (D) and no decrease in fluorescence lifetime could be observed. Bar, 10 µm.

Sequence analysis of AtPEN-2 revealed that a similar motif containing this crucial asparagine residue is well conserved (see Fig. 1D). To investigate its role in AtPEN-2 localization we conducted site-directed mutagenesis, substituting asparagine with leucine (N74L) and verified localization of the mutated form of AtPEN-2 in *A. thaliana* protoplasts (Fig. 6). Intriguingly, AtPEN-2^N74L^ signal was observed only in the reticular compartment (Fig. 6A, left), whereas the native AtPEN-2 localized to the vesicular compartment (Fig. 6A, right; see also Fig. 2K). We identified this compartment as ER by colocalization with the endoplasmic reticulum marker protein NIP1;1 (Fig. 6B). Moreover, mutated AtPEN-2^N74L^ no longer showed colocalization with the TGN marker (Fig. 6C) or PVC marker (not shown) (to compare see Fig. 3G, H). These data clearly suggest that loss of the conserved asparagine disturbed vesicular localization of AtPEN-2 protein.

In addition to a mislocalization of AtPEN-2^N74L^, we observed that AtPS2 is no longer re-localized when co-expressed with AtPEN-2^N74L^. When AtPS2 and native AtPEN-2 were co-expressed in *A. thaliana* protoplasts both proteins colocalized mainly to the vesicular compartment (see Fig. 5H). In contrast, when expressed with mutated AtPEN-2^N74L^, AtPS2 was restricted to the reticular compartment, with no visible localization to the vesicular compartment (Fig. 6D). We conducted FLIM measurements to check whether the interaction between AtPEN-2^N74L^ and AtPS2 was maintained. The fluorescence lifetime of the donor AtPEN-2^N74L^ fused with eGFP was measured in the absence (T_D_ = 2.36 ns) and in the presence of AtPS2–RFP (T_DA_ = 2.34 ns). The calculated FRET efficiency for this pair was very low (E=1%) clearly suggesting that no interaction occurs between AtPEN-2^N74L^ and AtPS2. These data indicate that in plants AtPEN-2 may be involved in presenilin trafficking. They also support a direct interaction between AtPEN-2 and AtPS2, although the molecular mechanism underlying these observations needs to be further investigated.

### An *A. thaliana* presenilin double mutant shows accelerated chlorosis upon dark treatment

To investigate the possible function of γ-secretase in *A. thaliana* we obtained single mutant insertion lines containing T-DNAs in the exons of presenilin1 (SALK_013158; *ps1*), and presenilin2 (SALK_145544; *ps2*). Homozygous mutant plants were identified and the absence of full-length transcript in each line was confirmed by RT-PCR (data not shown). Single presenilin mutants *ps1* and *ps2* lines as well as a double mutant in both presenilin genes (*ps1*/*ps2*) appeared indistinguishable from wild type under normal growth conditions.

**Fig. 7.**
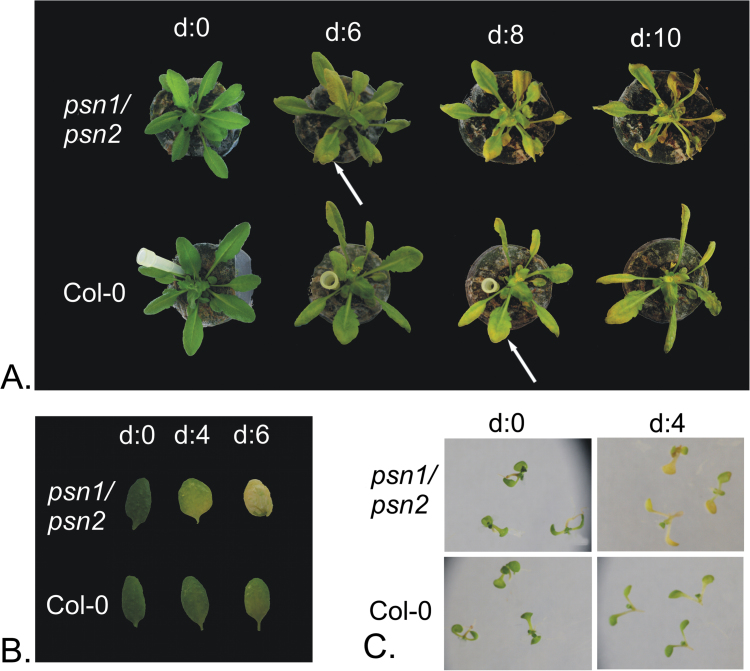
Presenilin double mutant plants show accelerated chlorosis upon darkness treatment. Leaves of plants from double mutant line *ps1/ps2* showed yellowing earlier compared with wild-type plants. This effect could be observed in 6-week-old plants (A), detached leaves (B), and seedlings (C).

Interestingly, in controlled dark conditions, leaves of 6-week-old double mutant *ps1*/*ps2* plants, showed accelerated yellowing 2 days earlier than wild type (Fig. 7A). Accelerated chlorosis could not be detected in either of single mutant lines for presenilin genes (data not shown). Detached leaf assay was conducted to confirm this observation. The second true leaves of 4-week-old rosettes were excised and floated on water. The leaves were placed in the dark and retrieved at various time points. Again chlorosis of double mutant leaves could be observed approximately 2 days earlier than in the wild type (Fig. 7B). The phenotype could be also observed in seedlings grown *in vitro*. Seeds were germinated on half-strength MS medium with 1.5% sucrose. At 2 weeks, seedlings were transferred to plates containing no nitrogen and no carbon (no N/C) medium, placed in the dark, and retrieved at various time points. Double mutant plants showed yellowing after only four days of darkness, when no signs of chlorosis were yet visible in wild-type plants (Fig. 7C).

**Fig. 8.**
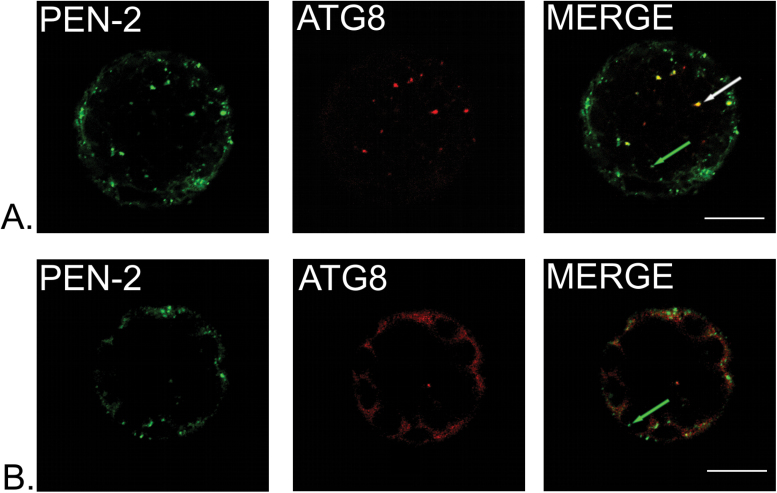
AtPEN-2 is partially localized to autophagosome vesicles in *Arabidopsis* protoplasts. (A) AtPEN-2 colocalizes with AtATG8, protein marker for autophagosome in plants (white arrow). However, a set of vesicles with AtPEN-2–GFP signal alone could be seen (green arrow) indicating existence of two populations of AtPEN-2 protein. (B) AtPEN-2 did not show colocalization when AtATG8 was not recruited to autophagosome membranes and signal was present in cytoplasm. Bar, 10 µm.

In plants, darkness and starvation are known to induce senescence and autophagy. Interestingly, mutants in some genes involved in autophagy displayed similar phenotypes to those described in *vti12* (extensively discussed in Surpin *et al.*, 2003) and *ps1*/*ps2* (our study). To investigate whether γ-secretase subunits could be somehow connected to autophagy, we tested whether AtPEN-2–GFP could colocalize with autophagy marker in *Arabidopsis* protoplasts. AtATG8 is a key molecular component required for the formation of autophagosomal membranes (Slavikova *et al.*, 2008; Nair *et al.*, 2012). Leaves for protoplasts isolation were pre-treated with darkness for 48h to induce autophagy. In approximately 80% of cells, clear colocalization of AtPEN-2 with large vesicular structures marked with AtATG8 fluorescence was observed (Fig. 8A), indicating that AtPEN-2 is localized to autophagosomes. A population of smaller vesicles marked with AtPEN-2 signal alone could also be observed. In 20% of protoplasts AtATG8 was not recruited to autophagosome membrane but dispersed in cytoplasm and showed no colocalization with AtPEN-2 protein.

## Discussion

Here we provide the first genetic and functional data on the γ-secretase complex in angiosperms. In a series of experiments, we show that homologues of γ-secretase subunits are not only present, but also highly conserved in *Arabidopsis*. Key amino acid motifs known from animals to be involved in catalysis or complex assembly are present and well conserved in plants (Fig. 1). Colocalization studies combined with FLIM-FRET analysis in *A. thaliana* protoplasts clearly indicate that particular γ-secretase subunits may interact with each other to form a complex (Fig. 5). Moreover, by means of site directed mutagenesis, we have identified an amino acid motif (WFVNCFYF) present in AtPEN-2 protein, which seems to be involved in interaction with presenilin 2 (Fig. 6). Collected data still do not allow us to build a model of γ-secretase assembly in plants. We believe, however, that subsequent mutational analysis combined with localization and interaction studies shall bring us in the future closer to resolving the γ-secretase complex topology in plants.

Our results clearly demonstrate that all γ-secretase subunits localize to compartments of the endomembrane system in plants. We have found that particular γ-secretase subunits localize to endoplasmic reticulum, the *trans* Golgi network (early endosome), and prevacuolar compartment (late endosome) (Fig. 3). These data are consistent with multiple animal studies (Annaert *et al.*, 1999; Pasternak *et al.*, 2003; Réchards *et al.*, 2003; Jutras *et al.*, 2005; Fukumori *et al.*, 2006; Dries and Yu, 2008; Fassler *et al.*, 2010; Smolarkiewicz *et al.*, 2013). Fluorescent aggregates may be the result of improper folding and/or trafficking of fusion protein, which would not be surprising considering the large number of transmembrane domains present in certain γ-secretase subunits. However, Phan *et al.* (2008) showed that formation of fluorescent aggregates of AtSNX2b, a sorting nexin, is not an artefact, but is a result of impaired overall vacuolar trafficking in response to nexin overexpression (Phan *et al.*, 2008). In this context, the next important question to ask will be whether γ-secretase subunits are just passive cargo, or whether as in animals they play a role in regulation of vesicle trafficking.

Data from *Physcomitrella* and *Dictyostelium* indicate that presenilin and γ-secretase are involved in endocytosis and phagocytosis, respectively (Khandelwal *et al.*, 2007; McMains *et al.*, 2010). In addition, a growing body of data shows that in animals γ-secretase or presenilin itself may be involved in the regulation of endocytosis of membrane proteins, their trafficking, and degradation (Zhang *et al.*, 2006; Repetto *et al.*, 2007; Tamboli *et al.*, 2008). This has led to the idea that γ-secretase, or presenilin itself, might originally have been involved in the degradation and recycling of membrane proteins (Lichtenthaler *et al.*, 2011), and that signalling functions associated with γ-secretase cleavage were acquired later in evolution (McMains *et al.*, 2010). Thus, the ancestral function of γ-secretase would have been to act as a ‘membrane proteasome’ (Kopan and Ilagan, 2004). This is consistent with the observation that presenilins may regulate cell signalling by targeting membrane proteins for degradation or endosomal recycling. Moreover, accumulating experimental data indicate a possible role for presenilins in phagocytic or autophagy-mediated lysosomal protein degradation (Neely *et al.*, 2011; Smolarkiewicz *et al.*, 2013). Localization of particular *Arabidopsis* γ-secretase subunits to a fraction of TGN and PVC may supports their possible function in these compartments and a potential involvement of γ-secretase in protein trafficking in *Arabidopsis*.

In our study we show that certain γ-secretase subunits colocalize with the VTI12 protein in *Arabidopsis* protoplasts (see Fig. 3E, G). VTI12 is a v-SNARE protein localized to some extent both in the TGN and PVC, and is involved in storage vacuole trafficking in *Arabidopsis* (Surpin *et al.*, 2003; Sanmartín *et al.*, 2007). Intriguingly, the *vti12* mutant described by Surpin *et al.* (2003) showed a very similar phenotype to the presenilin double mutant described here. In normal growth conditions *vti12* plants show no phenotype compared with wild type plants. However, in nutrition limited conditions and upon dark treatment, accelerated chlorosis of mutant plants could be observed (Surpin *et al.*, 2003). Data presented here may be considered as a starting point for further, more detailed analysis of γ-secretase and its possible role in plants.

One of the most important questions to address in the future is the nature of substrates of the γ-secretase complex in plants. None of over 80 γ-secretase substrates identified in animals has an obvious homologue in plants. Moreover, Khandelwal *et al.* (2007) were unable to detect moss γ-secretase activity against Notch-based substrates. This does not exclude, however, a possible role for γ-secretase in regulating intramembrane proteolysis in plants. It has been shown, for example that AtRBL12, an *Arabidopsis* rhomboid-like protease, which also belongs to I-CLiPs, does not recognize yeast substrates (Kmiec-Wisniewska *et al.*, 2008). However, a different rhomboid-like protease, AtRBL2, was shown to be active against a *Drosophila* substrate, Spitz, even though no Spitz orthologue is present in plants (Kanaoka *et al.*, 2005). In plants, more than 80 membrane-bound transcription factors are predicted. A few of these have been characterized, and shown to be processed by regulated intramembrane proteolysis (Slabaugh and Brandizzi, 2011). Gamma-secretase could be involved in these processing events. Alternatively, as there is now considerable evidence that in metazoans and in Dictyostelium the γ-secretase complex, or presenilin itself, may be involved in the proteolysis-independent regulation of signalling pathways (Ludtmann et al., 2014) or regulation of such processes as transport of membrane proteins or cell adhesion (Coen and Annaert, 2010), a similar mode of function could also be envisaged for plant γ-secretase. The variety of possible functions requires further and careful investigation. However, data provided in this study may be a starting point for a discussion of the possible roles of the γ-secretase complex in plants.
